# PET imaging of the mouse brain reveals a dynamic regulation of SERT density in a chronic stress model

**DOI:** 10.1038/s41398-019-0416-7

**Published:** 2019-02-11

**Authors:** Sonali N. Reisinger, Thomas Wanek, Oliver Langer, Daniela D. Pollak

**Affiliations:** 1 Department of Neurophysiology and Neuropharmacology, Center for Physiology and Pharmacology, Medical University of Vienna, Schwarzspanierstraße 17, 1090 Vienna, Austria; 2 Biomedical Systems, AIT Austrian Institute of Technology GmbH, 2444 Seibersdorf, Austria; 3 Department of Clinical Pharmacology, Medical University of Vienna, Währinger Gürtel 18-20, 1090 Vienna, Austria; 4 Department of Biomedical Imaging und Image-guided Therapy, Division of Nuclear Medicine, Medical University of Vienna, Währinger Gürtel 18-20, 1090 Vienna, Austria

## Abstract

The serotonin transporter (SERT, Slc6a4) plays an important role in the regulation of serotonergic neurotransmission and its aberrant expression has been linked to several psychiatric conditions. While SERT density has been proven to be amenable to in vivo quantitative evaluation by positron emission tomography (PET) in humans, this approach is in its infancy for rodents. Here we set out to evaluate the feasibility of using small-animal PET employing [^11^C]DASB ([^11^C]-3-amino-4-(2-dimethylaminomethyl-phenylsulfanyl)-benzonitrile) as a radiotracer to measure SERT density in designated areas of the mouse brain. Using *Slc6a4*^*+/+*^, *Slc6a4*^*+/−*^, and *Slc6a4*^*−/−*^ mice as a genetic model of different SERT expression levels, we showed the feasibility of SERT imaging in the mouse brain with [^11^C]DASB-PET. The PET analysis was complemented by an evaluation of SERT protein expression using western blot, which revealed a highly significant correlation between in vivo and ex vivo measurements. [^11^C]DASB-PET was then applied to the examination of dynamic changes of SERT levels in different brain areas in the chronic corticosterone mouse model of chronic stress. The observed significant reduction in SERT density in corticosterone-treated mice was independently validated by and correlated with western blot analysis. This is the first demonstration of a quantitative in vivo evaluation of SERT density in subregions of the mouse brain using [^11^C]DASB-PET. The evidenced decrease in SERT density in response to chronic corticosterone treatment adds a new dimension to the complex involvement of SERT in the pathophysiology of stress-induced mental illnesses.

## Introduction

The serotonergic neurotransmitter system comprises a diffuse neuronal network that is involved in the control of several fundamental brain functions, including the regulation of mood^1–3^, sleep/wake rhythms^4,5^, aggression^6^, appetite^7,8^, learning and memory, and reward^9–11^. Correspondingly, alterations in serotonergic neurotransmission are implicated in a wide range of mental illnesses, from mood and anxiety to substance abuse disorders^12,13^ and many psychoactive medications impact on the serotonergic system.

The activity and kinetics of serotonergic neurotransmission are critically dependent upon reuptake of serotonin (5-HT) from the synaptic cleft into the presynaptic neuron by the serotonin transporter (SERT, SLC6A4). Over the last four decades, its important role as a major regulatory element of the 5-HT system has made SERT an attractive drug target for the development of psychoactive medications. Indeed, some of the most widely used pharmacological therapies for mood and anxiety disorders act upon SERT. Additionally, SERT-gene polymorphisms and their relevance for sensory processing sensitivity^14^ have contributed to the notion of a complex involvement of SERT within the gene × environment interactions shaping the susceptibility for the development of psychiatric disorders^15–18^.

However, despite more than 40 years of intensive research efforts the specific role of SERT in mental illness and the therapeutic response to psychotropic medications remains incompletely understood^19,20^. Limitations in the translatability from preclinical research to the human patient have posed a major obstacle both in basic research and drug development^21,22^. Methodologies that are amenable to human application as well as animal model experiments hold the potential to aid in bridging the gap from bench to bedside. Positron emission tomography (PET) is one of these methods, which can measure the kinetics of radiolabeled molecules (so-called radiotracers) in virtually all tissues of the body in a minimally invasive manner. With the availability of a constantly increasing toolbox of radiotracers that specifically bind to a wide array of different molecular target structures, PET holds great potential to monitor disease progression and treatment response. In the last two decades, the development of small-animal PET technology has allowed the examination of humans and rodents in comparable paradigms, allowing for a minimally invasive in vivo investigation of neural circuitries and molecular targets in longitudinal studies in patients and experimental animals. For quantification of SERT density in the brain, [^11^C]DASB (3-amino-4-(2-dimethylaminomethylphenylsulfanyl)-benzonitrile)^23^ has been most successfully used as a radiotracer in human studies and is currently considered the gold standard in the field^24–26^. However, the implementation of SERT-PET imaging in the translational neurosciences using animal models is a challenging task, as reflected in the difficulties to obtain accurate quantitative parameters of SERT binding in the mouse brain using a PET-based approach^27^.

Here we set out to establish [^11^C]DASB-PET as a suitable tool for the in vivo monitoring of SERT density in the mouse brain with the aim to detect dynamic changes in SERT levels in response to environmental conditions favoring the development of psychiatric conditions in a validated animal model.

## Materials and methods

### Chemicals

Unless otherwise stated, all chemicals were purchased from Sigma-Aldrich (Schnelldorf, Germany) or Merck (Darmstadt, Germany).

### Radiotracer synthesis

[^11^C]DASB was synthesized by *N*-methylation of *N*-desmethyl-DASB (ABX GmbH, Radeberg, Germany) with [^11^C]methyl iodide as described in the literature^28,29^. The entire synthesis procedure was fully automated in a Tracerlab FX C Pro synthesis module (GE Healthcare Uppsala, Sweden). [^11^C]DASB was formulated in physiological saline solution (0.9%, w/v) containing 0.1% (v/v) polysorbate-80 and a maximum of 5% (v/v) ethanol for intravenous (i.v.) injection into animals. Radiochemical purity of [^11^C]DASB was >98%. Molar activity at the time of radiotracer injection into different animal groups is reported in Table 1.

**Table 1:** Overview of examined animals, groups, and numbers investigated with [^11^C]DASB-PET

Genotype	Number/sex	Body weight (g)	Injected activity (MBq)	Molar activity^a^ (GBq/µmol)	Injected dose (nmol/kg)
**Part A**					
Wild type (*Slc6a4*^*+/+*^)	8/male	24.9 ± 2.3	22.5 ± 18.8	210.6 ± 101.3	3.9 ± 2.6
	5/female	22.1 ± 2.3	40.4 ± 3.7	240.2 ± 173.5	12.6 ± 9.6
* Slc6a4* ^*+/–*^	5/male	25.8 ± 2.3	34.5 ± 5.1	331.4 ± 97.5	4.4 ± 1.5
	5/female	19.5 ± 1.5	37.6 ± 5.8	271.7 ± 96.4	7.8 ± 2.5
* Slc6a4* ^*−/–*^	5/male	25.7 ± 4.1	33.6 ± 4.7	261.0 ± 183.8	7.3 ± 4.1
	4/female	19.0 ± 1.8	35.5 ± 4.1	333.2 ± 331.7	10.3 ± 6.7
Wild type (*Slc6a4*^*+/+*^)	4/male	27.2 ± 1.2	29.0 ± 3.2	48.9 ± 10.4	152.8 ± 12.1
low molar activity					
**Part B**					
Wild type					
Baseline PET	28/male	23.8 ± 1.1	6.2 ± 1.6	73.2 ± 13.5	3.8 ± 1.7
Post-treatment PET					
CORT	20/male	28.9 ± 1.6	5.64 ± 0.91	75.5 ± 26.3	2.94 ± 1.20
CTRL	8/male	29.7 ± 2.3	5.24 ± 0.58	57.1 ± 14.6	3.34 ± 1.25

*Slc6a4* gene encoding for the serotonin transporter, CORT corticosterone treatment, CTRL control/vehicle treatment

^a^At the time of radiotracer injection into the animals

### Animals

Heterozygous founder pairs for the generation of wild-type (*Slc6a4*^*+/+*^), heterozygous (*Slc6a4*^*+/−*^), and homozygous (*Slc6a4*^*−/−*^) SERT knockout mice on a C57BL/6J genetic background were provided by Harald H. Sitte (Medical University of Vienna, Austria). Wild-type C57BL/6N mice used in the chronic corticosterone (CORT) paradigm were obtained from Charles River (Sulzfeld, Germany). For a summary of animal numbers used for PET scans in each part of this study see Table 1. All mice were single-housed for 1 week before the start of experiments, kept on a 12-h light-dark cycle, and had ad libitum access to food and water. At the time of the first PET scan, animals weighed 23.5 ± 2.7 g.

A habituation period of at least 1 week was scheduled after the transfer to the preclinical imaging facility (Austrian Institute of Technology; Seibersdorf, Austria) and before the animals were subjected to PET scans. The study was approved by the national authorities (Amt der Niederösterreichischen Landesregierung) and study procedures were in accordance with the European Communities Council Directive of September 22, 2010 (2010/63/EU).

### Experimental design

**Fig. 1:**
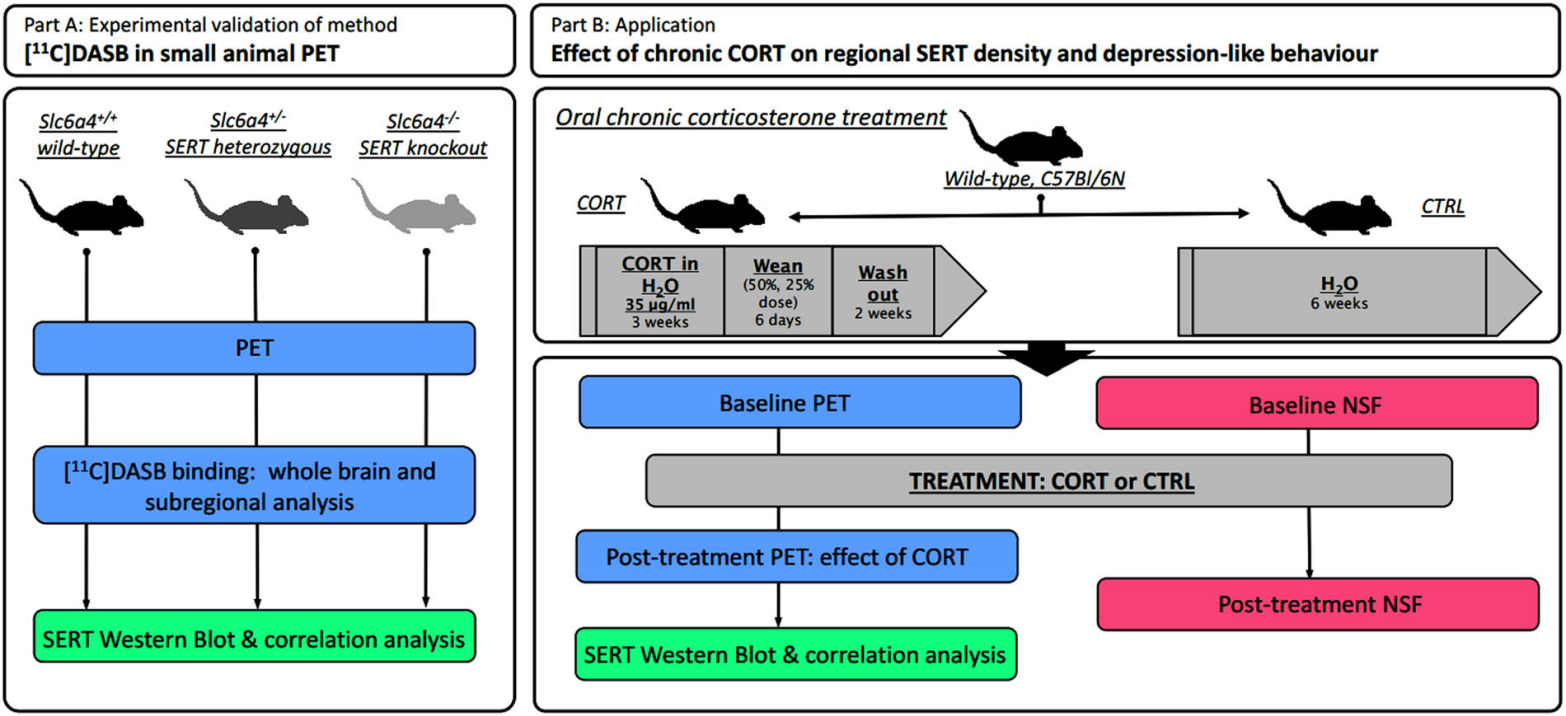
Experimental design. Schematic overview of the study: validation of [^11^C]DASB-PET using groups of *Slc6a4*^*+/+*^, *Slc6a4*^*+/−*^, and *Slc6a4*^−/*−*^ mice (part A) and investigation of the effects of a chronic CORT paradigm on SERT density as measured by [^11^C]DASB-PET and western blot, as well as depression-like behavior (part B). [^11^C]DASB [^11^C]-3-amino-4-(2-dimethylaminomethyl-phenylsulfanyl)-benzonitrile, PET positron emission tomography, Slc6a4/SERT serotonin transporter, NSF novelty-suppressed feeding test, CORT corticosterone treatment, CTRL control/vehicle treatment

An overview of the experimental design of this study is depicted in Fig. 1. In the first set of experiments (part A), cohorts of *Slc6a4*^*+/+*^, *Slc6a4*^*+/−*^, and *Slc6a4*^*−/−*^ mice underwent [^11^C]DASB-PET scans, followed by brain extraction and ex vivo determination of hippocampal SERT expression by western blot. In a second set of experiments (part B), wild-type mice underwent a baseline [^11^C]DASB-PET scan prior to being randomly assigned to either treatment with corticosterone (CORT group) or water (CTRL group) for 57 ± 2 days after which animals underwent a second [^11^C]DASB-PET scan. Subsequently, animals were sacrificed and post-treatment SERT expression was biochemically examined by western blot. A parallel cohort of mice was subjected to chronic CORT treatment and tested for depression-related anxiety using the novelty-suppressed feeding test (NSF) in order to validate the potential of the chronic CORT paradigm to induce depression-like behavior.

### Chronic corticosterone treatment

Mice were exposed to corticosterone-21-hemisuccinate (4-[2-[(8S,9S,10R,11S,13S,14S,17S)-11-hydroxy-10,13-dimethyl-3-oxo-1,2,6,7,8,9,11,12,14,15,16,17-dodecahydrocyclopenta[a]phen-anthren-17-yl]-2-oxoethoxy]-4-oxobutanoic acid; 35 μg/mL free base; Steraloids Inc., Newport, RI, USA) via the drinking water according to a published protocol^30^ with minor adaptations. Briefly, the CORT treatment lasted 3 weeks, followed by a weaning phase during which mice received a solution at 50% and then 25% of the full dose for 3 days respectively, with a subsequent washout period of 2 weeks to allow for the investigation of long-lasting effects of chronic CORT^30^. Control animals (CTRL) received regular drinking water for the entire duration of the experimental period.

### Novelty-suppressed feeding test

Mice (*n* = 7–8/group) were weighed and food-restricted 24 h prior to behavioral testing. On the day of testing, they were habituated to the experimental room for 45 min. A single food pellet was placed into the center of a brightly lit (800 lux) arena (30 × 50 cm) filled with wood-chip bedding material. The latency to feed on the pellet (s) was recorded for each mouse and used as a parameter to assess depression-related anxiety^31,32^. Mice that lost more than 20% of their body weight during the food restriction period were excluded from the analysis.

### PET imaging

Imaging experiments were performed under isoflurane anesthesia (1.5–3.0% in oxygen). Animals were warmed throughout the experiment and body temperature and respiratory rate were constantly monitored. Mice were placed in an imaging chamber and the lateral tail vein was cannulated for i.v. administrations. A microPET Focus220 scanner (Siemens Medical Solutions, Knoxville, TN, USA) was used for PET imaging. Dynamic emission scans (90 min) were started with the i.v. injection of [^11^C]DASB (see Table 1 for injected doses in individual animal groups). To assess the dose-dependency of SERT binding of [^11^C]DASB, unlabeled DASB (ABX GmbH, Radeberg, Germany) was co-injected with the radiotracer in a group of wild-type animals (*n* = 4, Table 1). List-mode data were acquired with a timing window of 6 ns and an energy window of 250–750 keV. For part A, at the end of the PET scan, a final blood sample (20–40 µL) was collected from the retro-orbital venous plexus and animals were sacrificed by cervical dislocation under deep anesthesia. Brains were extracted and blood was centrifuged (13,000 × *g*, 4 °C, 4 min) to obtain plasma. Aliquots of blood and plasma were transferred into pre-weighed tubes and measured for radioactivity in a gamma counter (Wizard 1470, PerkinElmer, Wellesley, MA, USA). The measured radioactivity data were corrected for radioactive decay and expressed as standardized uptake values ((radioactivity per g/injected radioactivity) × body weight). For part B, at the end of the baseline PET scan, a small blood sample (10–15 µL) was collected and animals were placed back into their cages for repeated [^11^C]DASB imaging following CORT or CTRL treatment. After the last PET scan, animals were sacrificed and processed as described above.

### Magnetic resonance imaging

Anatomical magnetic resonance (MR) imaging was performed prior to PET imaging on a 1 T benchtop MR scanner (ICON, Bruker BioSpin GmbH, Ettlingen Germany) in a subset of animals (*n* = 8). For MR imaging a modified three-dimensional (3D) T1-weighted gradient echo sequence (T_1_-FLASH) was used and the following imaging parameters: echo time = 4.7 ms; repetition time = 27 ms; flip angle = 25°; field of view = 7.6 × 2.6 × 2.4 cm; matrix = 380 × 130; 32 slices; slice thickness = 750 µm; and total imaging time = 22 min.

### PET data analysis

The dynamic PET data were sorted into 25 frames, which incrementally increased in time length. PET images were reconstructed using Fourier re-binning of the 3D sinograms followed by a two-dimensional-filtered back-projection with a ramp filter giving a voxel size of 0.4 × 0.4 × 0.796 mm^3^. Using PMOD software (version 3.6, PMOD Technologies Ltd., Zurich, Switzerland), hippocampus, striatum, thalamus, cortex, and cerebellum were outlined on the PET images using the Mirrione Mouse Atlas and guided by representative MR images obtained in a few animals. The position of the regions of interest (ROIs) was manually adjusted if necessary without modification in ROI sizes. Following ROI definition, time-activity curves (TACs) expressed in standardized uptake value units were derived. Regional PET data were modeled with the PKIN tool in PMOD using a simplified reference tissue model and whole cerebellum as a reference tissue to obtain the non-displaceable binding potential (BP_nd_) as an outcome parameter for SERT-specific binding of [^11^C]DASB^27^. BP_nd_ is defined as the ratio between the available SERT density (*B*_max_) and the apparent equilibrium dissociation constant (*K*_D_) of the radiotracer. Under the assumption that *K*_D_ remains unchanged, BP_nd_ can be considered as a measure of SERT density. To assess the dose-dependency of SERT-specific binding of [^11^C]DASB in wild-type (*Slc6a4*^*+/*+^) mice, a sigmoidal dose-response curve was fitted to a plot of BP_nd_ in the hippocampus, striatum, thalamus, and cortex to injected DASB dose (nmol/kg) using the following equation:

$${\mathrm{BP}}_{{\mathrm{nd}}} = {\mathrm{BP}}_{{\mathrm{nd}},\max }\frac{{{\mathrm{Dose}}}}{{\left( {{\mathrm{ED}}_{{\mathrm{50}}} + {\mathrm{Dose}}} \right)}}$$

where Dose is the injected DASB dose (nmol/kg), ED_50_ the half-maximum effect dose of DASB to displace SERT-specific binding of [^11^C]DASB (nmol/kg), and BP_nd,max_ is the maximum BP_nd_ value. A fixed Hill slope of −1.0 was assumed.

### Determination of reproducibility and reliability of [^11^C]DASB in CTRL group

Test-retest analysis was performed as described in the literature^33^ by comparing BP_nd_ values in different brain regions at baseline with BP_nd_ values of the CTRL group measured approximately 57 days after baseline (*n* = 8).

The test-retest variability (VAR) was calculated as follows:

$${\mathrm{VAR}} = \frac{{\left| {{\mathrm{scan}\,{2}} - {\mathrm{scan}\,{1}}} \right|}}{{0.5 \times \left( {{\mathrm{scan}\,{1}} + {\mathrm{scan}\,{2}}} \right)}}$$

where scan 2 represents the BP_nd_ values of the CTRL group and scan 1 represents the BP_nd_ values of the respective animals at baseline.

The test-retest reliability (RL) was calculated using the following formula:

$${\mathrm{RL}} = \frac{{{\mathrm{STDB}}^2}}{{\left( {{\mathrm{STDB}}^2 + {\mathrm{STDW}}^2} \right)}}$$

where STDB represents the standard deviation between the subjects and STDW represents the standard deviations within the subject at both imaging time points.

### Western blot

Immediately after the last PET scan, brains were rapidly extracted and the hippocampus, cerebellum, and cortex were dissected. Tissue samples were then snap-frozen in liquid nitrogen and stored at −80 °C until used for western blot analysis.

Total protein was extracted from powderized brain tissue and 50 µg of protein were loaded and subjected to SDS-polyacrylamide gel electrophoresis according to a previously described procedure^34^. Membranes were incubated overnight with the primary antibodies (SERT, target: sc-1458, dilution 1:500; secondary antibody: anti-goat IgG-HRP, sc-2020, dilution 1:2000; both Santa Cruz Biotechnology, Inc, Dallas, TX, USA; β-actin, housekeeping protein: A0760-40, dilution 1:2000; US Biological, Swampscott, MA, USA; secondary antibody: anti-mouse IgG-HRP, #7076, dilution 1:5000; Cell Signaling Technology, Danvers, MA, USA) used as the housekeeping protein. Chemiluminescent imaging was performed using a FluorChem HD2 imager (Alpha Innotec, Kasendorf, Germany) and densitometry values were determined using the software ImageJ (NIH, Bethesda, MD, USA). SERT protein densitometry values for each sample were normalized to the corresponding housekeeping protein value to obtain semiquantitative measures of SERT expression.

### Statistical analysis

Differences between two groups were analyzed with a two-sided *t*-test (with Welch correction where applicable) and between multiple groups with two-way or one-way analysis of variance with post hoc Tukey’s multiple comparison test using Prism 7 software (GraphPad Software, La Jolla, CA, USA). To assess correlations, the Pearson correlation coefficient *r* was calculated. The level of statistical significance was set to a *P* value of <0.05. All values are given as mean ± standard deviation.

## Results

In the present study we set out to examine dynamic changes in SERT density in the mouse brain using PET imaging with [^11^C]DASB in a mouse model of chronic stress based upon long-term treatment with CORT. As [^11^C]DASB had so far not been successfully used to monitor SERT density in the mouse brain in quantitative terms, we first conducted a comprehensive validation of [^11^C]DASB as a SERT-PET tracer in mice (see experimental design, Fig. 1, part A). To this end, we followed two different approaches. First, we examined SERT wild-type (*Slc6a4*^*+/+*^), heterozygous (*Slc6a4*^*+/−*^), and homozygous knockout (*Slc6a4*^*–/–*^) mice as genetic models of differential SERT expression. Second, as an additional validation step, we co-injected wild-type mice with unlabeled DASB to determine the ED_50_ for displacement of SERT-specific binding of [^11^C]DASB. We calculated the BP_nd_ as outcome parameter of SERT-specific binding of [^11^C]DASB using whole cerebellum as a SERT-free reference tissue^27^.

**Fig. 2:**
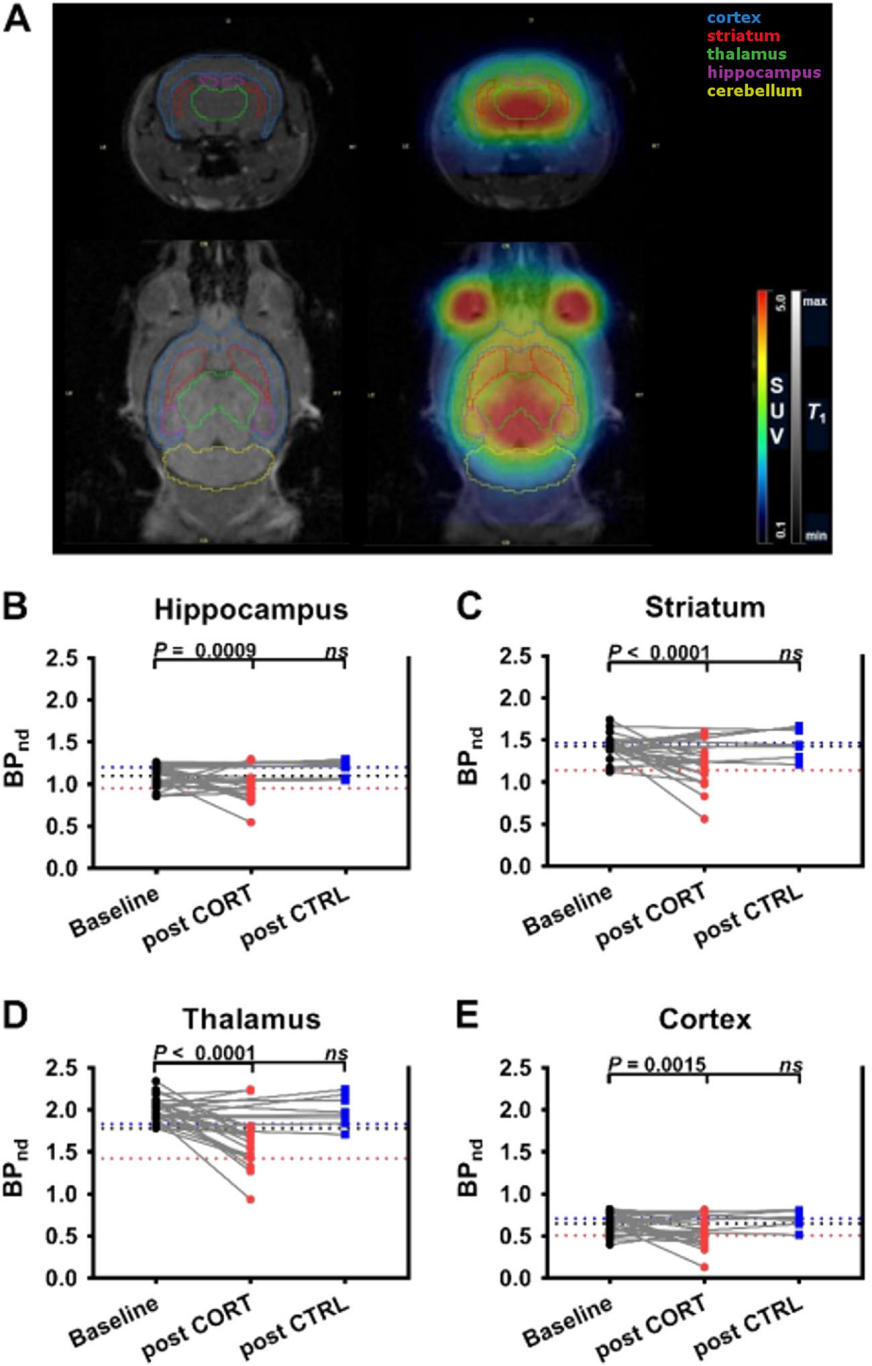
BP_nd_ values for CORT-treated and control groups. **a** Definition of brain ROIs (hippocampus—purple; striatum—red; thalamus—green; cortex—blue; cerebellum—yellow) for subregional analysis of SERT density on representative coronal and horizontal MR and MR co-registered PET summation images (0–90 min). BP_nd_ values at baseline and after treatment with corticosterone (CORT group) or vehicle (CTRL group) in **b** hippocampus, **c** striatum, **d** thalamus, and **e** cortex of wild-type mice. Black dotted line indicates mean BP_nd_ value of baseline scans, red dotted line indicates mean BP_nd_ value of second scan in CORT group, and blue dotted line indicates mean BP_nd_ value of second scan in CRTL group. One-way analysis of variance indicated a significant change in baseline BP_nd_ values following CORT treatment in hippocampus (*F*_2,52_ = 12.58, *P* < 0.0001), striatum (*F*_2,52_ = 16.82, *P* < 0.0001), thalamus (*F*_2,52_ = 20.51, *P* < 0.0001), and cortex (*F*_2,52_ = 10.03, *P* = 0.0002), *n* = 8–27. *P* values indicate results of post hoc Tukey’s multiple comparison analysis. Data are presented as paired individual values (gray lines) at baseline and after CORT or CTRL treatment. BP_nd_ non-displaceable binding potential, SUV standardized uptake value, ROI region of interest, CORT corticosterone treatment, CTRL control/vehicle treatment, ns nonsignificant, MR magnetic resonance, PET positron emission tomography

Considering the perspective of the application of [^11^C]DASB-PET for the evaluation of mouse models of neuropsychiatric disorders we strove to apply our analysis to the level of individual brain areas, by defining ROIs with relevance for SERT-related mental disorders. To this end, we used a mouse brain atlas in PMOD to outline the hippocampus, striatum, thalamus, and the cortex on PET images with the help of MR scans acquired in representative animals (Fig. 2a). TACs in these brain regions for the three mouse genotypes revealed clear visual differences, with highest values in *Slc6a4*^*+/+*^, followed by *Slc6a4*^*+/–*^ and *Slc6a4*^*−/–*^ mice in the SERT-rich brain regions hippocampus, striatum, and thalamus (Supplementary Figure S1A-C). These differences were less pronounced in the cortex (Supplementary Figure S1D), which contains considerably lower SERT expression levels than the other three brain regions^35^. TACs of the three genotypes were almost superimposable in the cerebellum, designated as reference tissue, indicating very low SERT expression levels (Supplementary Figure S1E). BP_nd_ values in different brain regions for different mouse genotypes are summarized in Supplementary Table S1. No sex differences in BP_nd_ values were observed for any of the brain regions examined in the three genotypes (Supplementary Figure S2A-D). In all brain regions, BP_nd_ values of male mice were significantly lower in *Slc6a4*^*−/−*^ and in *Slc6a4*^*+/−*^ mice compared to *Slc6a4*^*+/+*^ mice. For female animals, significant differences between the three genotypes were found for hippocampus, striatum, and thalamus, but not for the cortex, which was most likely due to the weak SERT expression in this brain region making it difficult to assess variations in [^11^C]DASB binding.

Blood concentration of radioactivity at the end of the PET scans was neither affected by genotype nor sex (Supplementary Figure S3).

This analysis was complemented by the examination of the dependency of BP_nd_ values on administered DASB dose (wild-type mice co-injected with unlabeled DASB) in all four brain regions (Supplementary Figure S4). The ED_50_ values of DASB to displace SERT-specific binding of [^11^C]DASB in the different brain regions are given in Supplementary Table S2. For the thalamus, the ED_50_ value (17.1 ± 1.4 nmol/kg) was in good agreement with the same value determined in a previous study in rats using cerebellar cortex as a reference tissue (12.0 ± 2.6 nmol/kg)^27^.

We next sought to additionally validate the PET-based determination of SERT density by an independent method. We therefore resorted to ex vivo analysis by western blotting of the same mouse brains analyzed by PET. We specifically focused on the hippocampus, in light of its involvement in chronic stress and the pathophysiology of several SERT-related mental disorders^36–38^. A highly significant positive correlation of BP_nd_ values with relative SERT expression levels determined by western blot was revealed (Supplementary Figure S5), further corroborating the specificity of the PET-based evaluation of SERT density and validating its experimental usage for longitudinal studies in mice.

Having established and validated [^11^C]DASB-PET as a method for the quantitative assessment of SERT density in individual regions of the mouse brain, we moved on to its first application: the examination of dynamic changes of brain SERT levels in the chronic CORT mouse model of chronic stress. To this end, mice were either subjected to a long-term oral CORT treatment (“chronic stress”) or received regular drinking water (CTRL). A parallel cohort of animals was used to verify the effect of CORT treatment on depression-related anxiety in the NSF^39^. We opted to not use the same animals in behavioral and imaging experiments in order to avoid effects of behavioral testing on SERT density. The animals of the imaging cohorts were scanned before (baseline) and after CORT and CTRL treatment, respectively (see experimental design, Fig. 1, part B). BP_nd_ values for the two PET scans in all four brain regions examined (Fig. 2a; hippocampus, striatum, thalamus, and cortex) revealed a significant decrease as compared to baseline in the CORT group but not in the CTRL group (Fig. 2b–e, Table 2). The mean percentage changes as compared to baseline scans in the CORT group were −9.3 ± 22.4% for the hippocampus, –19.7 ± 18.6% for the striatum, −20.6 ± 14.4% for the thalamus, and −15.4 ± 32.6% for the cortex. In 4 out of 20 animals of the CORT group BP_nd_ values were not decreased after CORT treatment but either remained similar or were slightly increased relative to baseline (Fig. 2b–e). CORT treatment did not affect blood radioactivity concentrations compared to baseline or CTRL (Supplementary Figure S6). We used the data acquired in the CTRL group to determine the test-retest VAR and RL of [^11^C]DASB-PET in different brain regions (see Supplementary Table S3).

**Table 2:** Quantification of [^11^C]DASB binding in brain regions of male wild-type mice at baseline (*n* = 28) and after CORT (*n* = 20) or CTRL (*n* = 8) treatment using a simplified reference tissue model with the whole cerebellum as reference tissue

Region	BP_nd_ ± SD	BP_nd_ ± SD	BP_nd_ ± SD
Part B	Baseline	CORT	CTRL
Hippocampus	1.10 ± 0.12	0.95 ± 0.17^a^	1.20 ± 0.09
Striatum	1.43 ± 0.14	1.14 ± 0.24^a^	1.47 ± 0.17
Thalamus	2.01 ± 0.15	1.61 ± 0.30^a^	1.99 ± 0.18
Cortex	0.65 ± 0.12	0.51 ± 0.15^a^	0.71 ± 0.10

*BP*_*nd*_ non-displaceable binding potential, *CORT* corticosterone treatment, *CTRL* control/vehicle treatment, *SD* standard deviation

^a^*P* ≤ 0.0002, one-way analysis of variance with Tukey’s multiple comparison test

**Fig. 3:**
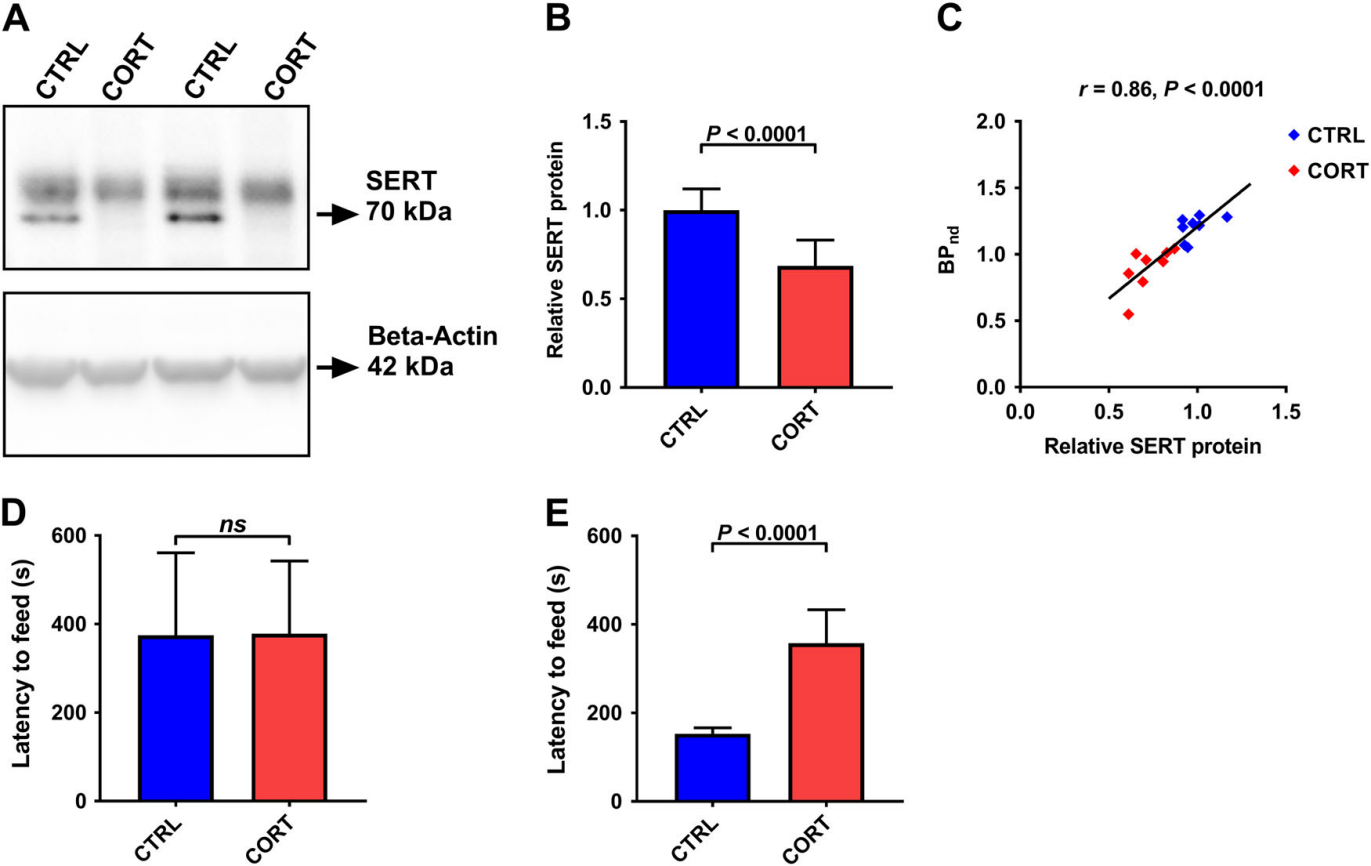
Effect of CORT on hippocampal SERT levels and depression-like behavior. **a** Representative western blot bands and **b** quantification of relative SERT protein in hippocampal tissue of wild-type mice of the CORT and CTRL groups (Welch corrected *t*-test: *t*_15.56_ = 5.095, *P* < 0.0001, *n* = 9–10). **c** Correlation between BP_nd_ values measured with PET and relative SERT protein expression assessed by western blot (Pearson correlation: *r* = 0.86, *P* < 0.0001, *n* = 17). Depression-like behavior in the NSF **d** prior to (Welch corrected *t*-test: *t*_13_ = 0.041, *P* = 0.968, *n* = 7–8) and **e** after (Welch corrected *t*-test: *t*_7.58_ = 7.534, *P* < 0.0001, *n* = 7–8) chronic CORT and CTRL treatments. Data are presented as mean ± SD. SERT serotonin transporter, CORT corticosterone treatment, CTRL control/vehicle treatment, BP_nd_ non-displaceable binding potential, NSF novelty-suppressed feeding test, ns nonsignificant, PET positron emission tomography

Western blot analysis revealed a significant difference in SERT expression between groups post treatment (Fig. 3a, b). We additionally correlated hippocampal BP_nd_ values measured in the second PET scan with SERT levels determined by western blot and found a significant positive correlation between the outcome measures obtained by the two independent methodologies (Fig. 3c). In the behavioral cohort, both groups showed similar levels of depression-related anxiety at baseline, as assessed by the latency to start feeding in the NSF. However, as expected upon treatment^39^, CORT animals presented with a significantly augmented latency to retrieve the food pellet in the NSF in comparison to controls, in which the post-treatment latency was shorter than the pre-treatment time to feed in the NSF possibly due to a degree of habituation to the testing apparatus and conditions (Fig. 3e).

## Discussion

We here demonstrate [^11^C]DASB-PET as a suitable and reliable method for the quantitative assessment of SERT density in selected regions of the mouse brain. We provide evidence for the application of the established approach in a longitudinal study design in a mouse model to demonstrate that the development of a behavioral phenotype associated with depression is paralleled by a dynamic regulation of SERT expression in several target brain areas. Previously it has been reported that [^11^C]DASB-PET can be used to determine SERT density in the rat brain, while the application to similarly accurate quantitative measurements in the mouse was considered difficult, most likely due to excessive levels of SERT occupancy induced by the injected mass of unlabeled DASB^27^. Here we met these challenges and report that by using [^11^C]DASB with moderately improved molar activity (Table 1) when compared to the study by Walker et al.^27^, as well as larger group sizes, we were able to measure previously reported genetic differences in SERT levels in the mouse brain^40^ and to validate in vivo PET measurements by independent, ex vivo biochemical assessments.

Working within the limitations of the low spatial resolution of PET, we were able to define individual serotonergic projection brain areas with relevance for neuropsychiatric disorders related to serotonergic neurotransmission and SERT function^40^. However, future technological improvements in terms of image reconstruction algorithms and analyses will be needed in order to include the evaluation of additional brain regions, which may be important when determining SERT density and its dynamic changes in the mouse. In particular, the midbrain (containing the dorsal raphe nucleus, a central area of origin of serotonergic cell bodies) could not be analyzed in the present study as the dimensions of this area in the mouse is far below the spatial resolution of our PET scanner (approximately 1.4 mm)^41^.

Although a higher degree of individual variability in SERT levels was observed mainly among male animals, no sex differences were found in the initial characterization of [^11^C]DASB in SERT wild-type and heterozygous mice. The regional analysis reflected the expected distribution pattern of SERT, with the highest [^11^C]DASB binding in the thalamus and striatum, followed by the hippocampus and lower binding levels in the cortex^35^. We noted only minor variations in [^11^C]DASB uptake in the cerebellum among different mouse genotypes (Supplementary Figure S1E), which suggested very low SERT expression levels in this reference tissue region^32^. This problem could be partly overcome in rats by using cerebellar cortex instead of whole cerebellum as a reference tissue^32^, which was, however, not possible in mice due to the inherent limitations of size and resolution. Therefore, a low level of SERT-specific binding of [^11^C]DASB in the cerebellum may have introduced a bias into the analysis of our mouse PET data. However, the excellent correlation between PET-derived BP_nd_ values and SERT density measured with western blot (Supplementary Figure S5C) suggested that our methodology was suitable to quantify SERT in vivo.

The central advantage of small-animal imaging over ex vivo analyses is the possibility for longitudinal evaluations in the form of multiple measurements in the same individual allowing for the assessment of the effects of chronic interventions/environmental conditions as well as aging over time. Here we benefitted from this possibility and addressed the dynamic impact of long-term exposure to chronic CORT (mimicking chronic stress as a precipitating factor for the development of several psychiatric disorders, including depression^42^), on SERT density within an individual mouse. Notably, the repeated-measures design allows for the detection of small, but significant treatment effects—such as the ones seen here—which might escape detection in traditional between-group designs employing ex vivo analysis of brain tissue. In the context of research into the physiological effects of stress, this becomes particularly relevant: individuals can display susceptibility or resilience to different stressors^43,44^, which was also observed in this study after CORT treatment (Fig. 2b–e). This may additionally mask small effects when using traditional study designs that do not include multiple measurements in a single animal.

Hence, we were able to reveal significant reductions in SERT density in the hippocampus, striatum, thalamus, and cortex after chronic CORT treatment and corroborated previous observations reporting individual behavioral responsivities upon exposure to various chronic stress paradigms^45,46^. The PET outcome parameter BP_nd_ is defined as the ratio between the available SERT density (*B*_max_) and the *K*_D_ of the radiotracer. The change in BP_nd_ caused by CORT treatment could thus either be due to changes in either *B*_max_ or *K*_D_, which we were not able to distinguish in the PET analysis approach applied herein. However, as our western blot data (Fig. 3a, b) revealed a significant reduction in SERT expression after CORT treatment, it can be assumed that the observed reduction in BP_nd_ primarily reflected a reduction in SERT density. A small number of studies investigating the effects of chronic CORT treatment on SERT expression in ex vivo analysis of brain tissue are available. These reports provide evidence for increases^47^ as well as decreases of SERT brain levels^48^ using different CORT administration protocols. We have recently described a dramatic reduction of SERT mRNA levels in the dorsal raphe nucleus^49^ in a CORT paradigm comparable to the one applied in the present study. Other studies using a variety of different stress protocols including chronic mild stress, social defeat, and chronic restraint stress have also examined the impact on SERT expression with diverging results^50–56^ possibly due to different rodent species used and methodological differences in the different chronic stress paradigms and CORT administration protocols. However, this variation in reported results is not exclusive to animal research^57^. One may speculate that the fact that inter-study variability is so high in this field may indicate a particular fragility—or indeed a particularly high degree of dynamic regulation—of the serotonergic system, and by consequence its major component SERT.

In conclusion, the present study fills an important gap, as it constitutes the first longitudinal examination of dynamic changes in SERT density in a mouse model of chronic stress. Future studies looking at SERT density at several time points during and after CORT treatment (or other chronic stress paradigms) will certainly give additional insight into the time course of SERT regulation in this context and aid to shed further light on the role of SERT in the development of stress-induced mental illnesses.

## Supplementary information

Supplementary Figure Legends and Tables_Clean

Supplementary Figure S1

Supplementary Figure S2

Supplementary Figure S3

Supplementary Figure S4

Supplementary Figure S5

Supplementary Figure S6
