# Supplementary material for: PET imaging of the mouse brain reveals a dynamic regulation of SERT density in a chronic stress model
Source: Transl Psychiatry. 2019 Feb 11;9:80. doi: 10.1038/s41398-019-0416-7 (PMC6370816; doi:10.1038/s41398-019-0416-7)
Supplement: Supplementary file 1 — Supplementary Figure Legends and Tables_Clean [file 41398_2019_416_MOESM1_ESM.docx]

**Supplementary Materials**

**Supplementary Tables**

**Table S1.** Quantification of [^11^C]DASB binding in brain regions of male and female wild-type (Slc6a4^+/+^), Slc6a4^+/-^ and Slc6a4^-/-^ mice using a simplified reference tissue model with the whole cerebellum as reference tissue. *Slc6a4*: serotonin transporter; BP_nd_: non-displaceable binding potential.

| **Genotype** | **Region** | **BP_nd_ ± SD** | **BP_nd_ ± SD** |
| --- | --- | --- | --- |
| **Part A** |  | male | female |
| wild-type  (*Slc6a4^+/+^*) | Hippocampus | 1.06±0.27 | 0.76±0.14 |
|  | Striatum | 1.55±0.26 | 1.14±0.21 |
|  | Thalamus | 2.12±0.29 | 1.58±0.31 |
|  | Cortex | 0.67±0.21 | 0.41±0.10 |
| *Slc6a4^+/-^* | Hippocampus | 0.59±0.09 | 0.57±0.03 |
|  | Striatum | 0.79±0.14 | 0.81±0.11 |
|  | Thalamus | 1.10±0.14 | 1.15±0.07 |
|  | Cortex | 0.30±0.09 | 0.27±0.07 |
| *Slc6a4^-/-^* | Hippocampus | 0.23±0.05 | 0.19±0.05 |
|  | Striatum | 0.30±0.03 | 0.27±0.17 |
|  | Thalamus | 0.34±0.16 | 0.25±0.18 |
|  | Cortex | 0.13±0.06 | 0.25±0.06 |
| wild-type (*Slc6a4^+/+^*)  low molar activity | Hippocampus | 0.29±0.05 | - |
|  | Striatum | 0.41±0.08 | - |
|  | Thalamus | 0.55±0.11 | - |
|  | Cortex | 0.14±0.03 | - |

**Table S2:** Estimated ED_50_ values for brain regions of male and female wild-type (*Slc6a4^+/+^*) mice (n=17) using nonlinear regression analysis of BP_nd_ values plotted as a function of the injected DASB dose. ED_50_: median effective dose; *R*^2^: coefficient of determination.

| **Region** | **ED_50_**  **(nmol/kg)** | ***R^2^*** |
| --- | --- | --- |
| Hippocampus | 4.8±1.4 | 0.88 |
| Striatum | 17.0±1.6 | 0.93 |
| Thalamus | 17.1±1.4 | 0.93 |
| Cortex | 3.9±2.1 | 0.73 |

**Table S3:** Test-retest variability and reliability of [^11^C]DASB in brain regions at baseline and approximately 57 days later, after CTRL treatment in male wild-type mice (n = 8). CTRL: control/vehicle treatment.

| **Region** | **Variability**  **(%)** | **Reliability**  **(%)** |
| --- | --- | --- |
| Hippocampus | 2.7±1.9 | 92.3±10.2 |
| Striatum | 7.2±5.7 | 80.9±21.3 |
| Thalamus | 4.5±4.7 | 81.9±24.6 |
| Cortex | 12.6±8.6 | 75.2±19.8 |

**Supplementary Figure Legends**

**Figure S1**. **Regional PET time-activity curves.**

Time-activity curves in in (A) hippocampus, (B) striatum, (C) thalamus, (D) cortex and (E) cerebellum of *Slc6a4^+/+^*, *Slc6a4^+/-^* and *Slc6a4^-/-^* mice. Cerebellum was used as a reference tissue with very low SERT expression levels in the PET data analysis. Data are presented as mean ± SD. *Slc6a4:* serotonin transporter; SUV: standardized uptake value.

**Figure S2**. **BP_nd_ values in different brain regions.**

BP_nd_ values determined with simplified reference tissue model in male and female *Slc6a4^+/+^*, *Slc6a4^+/-^* and *Slc6a4^-/-^* mice (n = 3-8 /group) in (A) hippocampus (2-way ANOVA, genotype: F_2,24_ = 53.91, *P* < 0.0001; sex: F_1,24_ = 2.07, *P* = 0.16; ), (B) striatum (2-way ANOVA, genotype: F_2,24_ = 89.34, *P* < 0.0001; sex: F_1,24_ = 2.28, *P* = 0.14), (C) thalamus (2-way ANOVA, genotype: F_2,23_ = 137.70, *P* < 0.0001; sex: F_1,23_ = 1.52, *P* = 0.23) and (D) cortex (2-way ANOVA, genotype: F_2,23_ = 15.71, *P* < 0.0001; sex: F_1,23_ = 1.14, *P* = 0.30). Cerebellum was used as a reference tissue with very low SERT expression levels in the PET data analysis. *P* values indicate results of *post hoc* Tukey’s multiple comparison analysis (*ns*: not significant). Individual BP_nd_ values with means ± SD are shown. *Slc6a4:* serotonin transporter; BP_nd_: non-displaceable binding potential.

**Figure S3**. **Blood radioactivity concentration values at 90 minutes after [^11^C]DASB administration**.

Measured blood radioactivity concentration values (SUV) at 90 minutes after intravenous [^11^C]DASB administration in male and female *Slc6a4^+/+^*, *Slc6a4^+/-^* and *Slc6a4^-/-^* mice (2-way ANOVA, genotype: F_2,27_ = 3.23, *P* = 0.06; sex: F_1,27_ = 3.32, *P* = 0.08; n = 4-8 /group). *P* values indicate results of *post hoc* Tukey’s multiple comparison analysis. Individual values with means ± SD are shown. *Slc6a4:* serotonin transporter; SUV: standardized uptake value; *ns*: non-significant.

**Figure S4**. **Dose dependency of [^11^C]DASB binding.**

Plot of BP_nd_ values (n = 17) determined with simplified reference tissue model using cerebellum as reference tissue *versus* injected DASB dose (nmol/kg) in (A) hippocampus, (B) striatum, (C) thalamus and (D) cortex of wild-type mice. Data were fitted to a sigmoidal model (broken line). See Supplementary Table S2 for estimated ED_50_ values in individual regions. BP_nd_: non-displaceable binding potential; ED_50_: median effective dose; *R*^2^: coefficient of determination.

**Figure S5**. **Correlation of PET data with Western blot data.**

(A) Representative images of hippocampal SERT Western blot for *Slc6a4^+/+^*, *Slc6a4^+/-^* and *Slc6a4^-/-^* mice and (B) corresponding quantification (1-way ANOVA, F_2,11_ = 97.58, *P* < 0.0001; n = 4-6 /group) *P*-Values indicate results of *post hoc* Tukey’s multiple comparison analysis. Data shows means ± SD. (C) Correlation between BP_nd_ values (n = 12) measured with PET and SERT/Actin ratio measured with Western blot in the hippocampus of *Slc6a4^+/+^*, *Slc6a4^+/-^* and *Slc6a4^-/-^* mice (*P* = 0.0001, *r* = 0.882). *Slc6a4:* serotonin transporter; BP_nd_: non-displaceable binding potential; *r* : Pearson correlation coefficient.

**Figure S6**. **Blood concentration of [^11^C]DASB for CORT-treated and control groups**

Blood concentration values at baseline and after CORT or CTRL treatment of male wild-type mice (1-way ANOVA, F_2,41_ = 0.10, *P* = 0.90; n = 8-20 /group). *P* Values indicate results of *post hoc* Tukey’s multiple comparison analysis. Data shows individual values with means ± SD. CORT: corticosterone treatment; CTRL: control/vehicle treatment; SUV: standardized uptake value; ns: non-significant.
