# Supplementary figures and images for: PET imaging of the mouse brain reveals a dynamic regulation of SERT density in a chronic stress model
Source: Transl Psychiatry. 2019 Feb 11;9:80. doi: 10.1038/s41398-019-0416-7 (PMC6370816; doi:10.1038/s41398-019-0416-7)

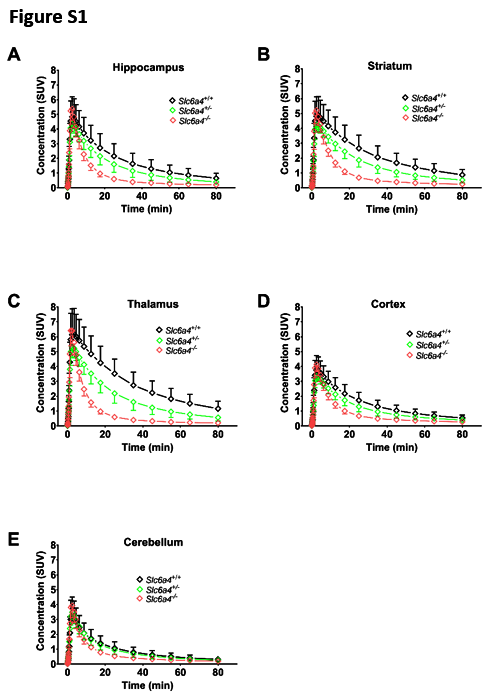

Supplement: Supplementary file 2 — Supplementary Figure S1 [file 41398_2019_416_MOESM2_ESM.tif]

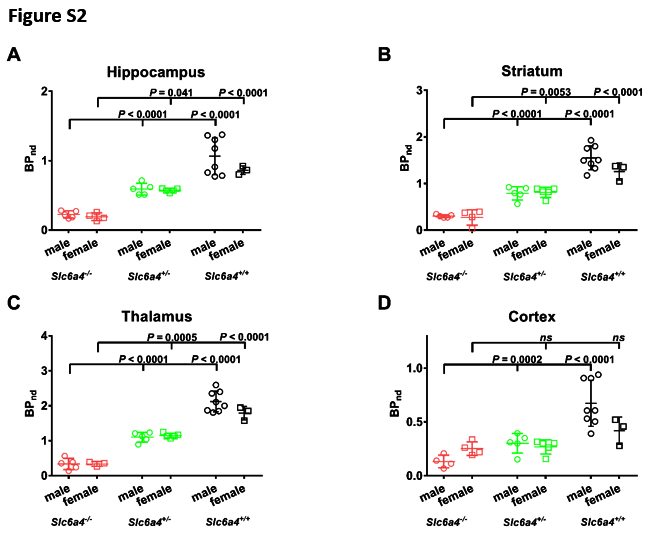

Supplement: Supplementary file 3 — Supplementary Figure S2 [file 41398_2019_416_MOESM3_ESM.tif]

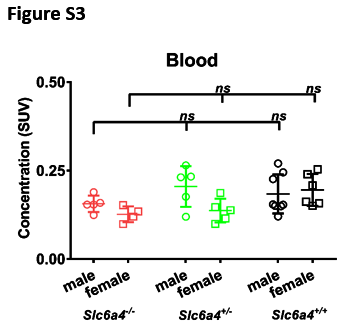

Supplement: Supplementary file 4 — Supplementary Figure S3 [file 41398_2019_416_MOESM4_ESM.tif]

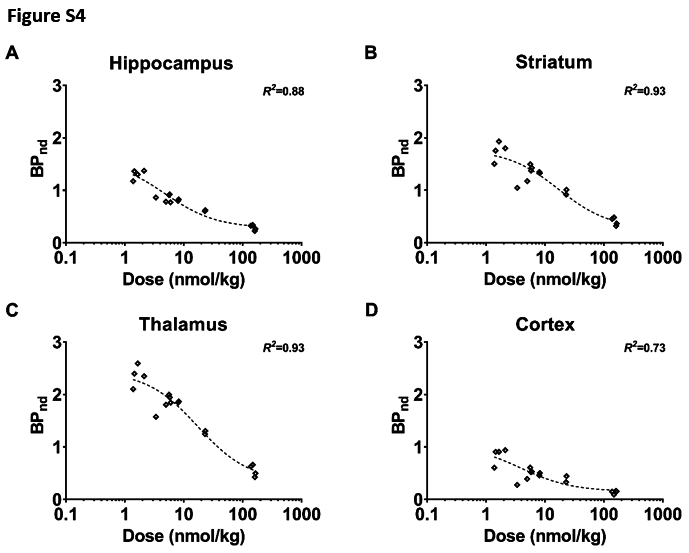

Supplement: Supplementary file 5 — Supplementary Figure S4 [file 41398_2019_416_MOESM5_ESM.tif]

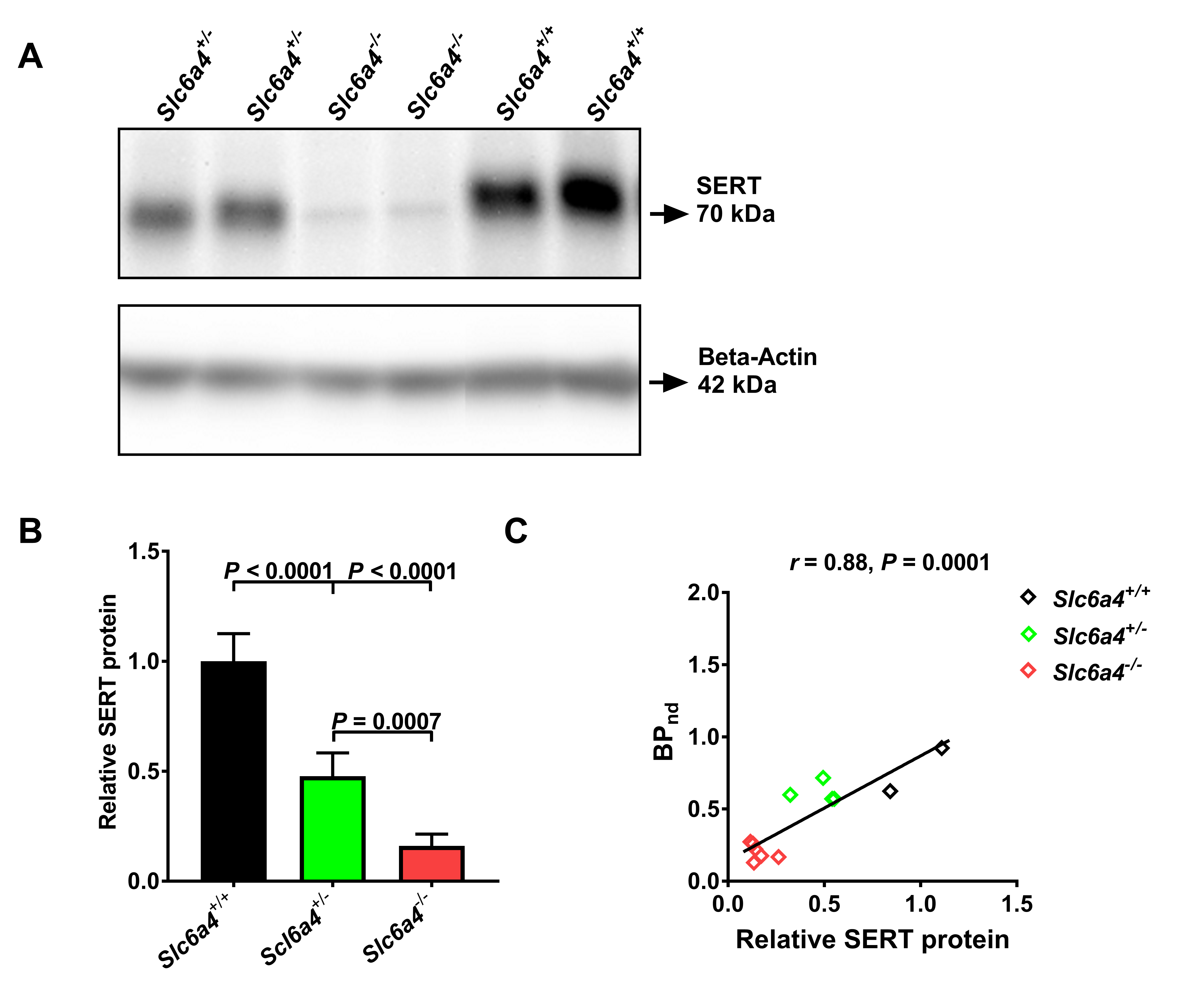

Supplement: Supplementary file 6 — Supplementary Figure S5 [file 41398_2019_416_MOESM6_ESM.tif]

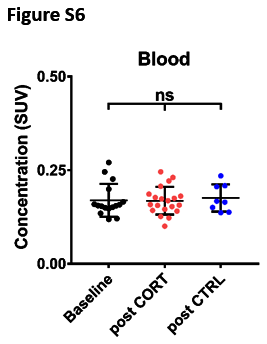

Supplement: Supplementary file 7 — Supplementary Figure S6 [file 41398_2019_416_MOESM7_ESM.tif]
